# Effects of polymorphisms in vitamin E-, vitamin C-, and glutathione peroxidase-related genes on serum biomarkers and associations with glaucoma

**Published:** 2013-02-03

**Authors:** Vicente Zanon-Moreno, Eva M. Asensio-Marquez, Lucia Ciancotti-Oliver, Jose J. Garcia-Medina, Pedro Sanz, Carolina Ortega-Azorin, Maria D. Pinazo-Duran, Jose M. Ordovás, Dolores Corella

**Affiliations:** 1 Genetic & Molecular Epidemiology Unit, Department of Preventive Medicine & Public Health, School of Medicine, University of Valencia, Valencia, Spain; 2 CIBER Fisiopatología de la Obesidad y Nutrición, University of Valencia, Valencia, Spain; 3 Department of Preventive Medicine & Public Health, Dr. Peset University Hospital, Valencia, Spain; 4 Department of Ophthalmology, Reina Sofia University General Hospital, Murcia, Spain; 5 Ophthalmology Research Unit “Santiago Grisolia,” Dr. Peset University Hospital, Valencia, Spain; 6 Nutrition and Genomics Laboratory, JM-USDA Human Nutrition Research Center on Aging at Tufts University, Boston, MA; 7 Department of Cardiovascular Epidemiology and Population Genetics, Centro Nacional de Investigaciones Cardiovasculares (CNIC), Madrid; 8 Instituto Madrileño de Estudios Avanzados (IMDEA) Alimentación, Madrid, Spain

## Abstract

**Purpose:**

To study the association of selected polymorphism in genes related to vitamin E, vitamin C, and glutathione peroxidase with these biomarkers and primary open-angle glaucoma (POAG) risk.

**Methods:**

A case-control study matched for age, sex, and bodyweight was undertaken. Two hundred fifty POAG cases and 250 controls were recruited from a Mediterranean population. Plasma concentrations of vitamin C, vitamin E, and glutathione peroxidase (GPx) activity were measured. We analyzed the polymorphisms rs1279683 in the Na^+^-dependent L-ascorbic acid transporter 2 (*SLC23A2*) gene, rs6994076 in the tocopherol alpha transfer protein (*TTPA*) gene, rs737723 in the tocopherol-associated protein (*SEC14L2/TAP*) gene, and rs757228 in the glutathione peroxidase 4 (*GPX4*) gene. We also analyzed expression of the *SLC23A2* gene in a subsample.

**Results:**

We found a novel association between the rs737723 polymorphism and POAG risk. Homozygous subjects for the C allele had a higher POAG risk than carriers of the ancestral G allele (adjusted odds ratio 1.73, 95% confidence interval 1.13–2.65, p=0.011). This association remained statistically significant after adjustment for multiple comparisons. We also confirmed the association between the rs1279683 polymorphism and a higher POAG risk in GG homozygous subjects and detected statistically significant differences in *SLC23A2* gene expression between POAG cases and controls, even after adjustment for multiple testing. We observed a nominally significant (p<0.05) gene–gene interaction between the *SEC14L2/TAP* and *SLC23A2* polymorphisms in determining POAG risk, increasing POAG risk in those subjects who had both risk genotypes at the same time (p<0.01). This increase was statistically significant even after adjustment for multiple comparisons. We did not detect any association with POAG risk for the rs6994076 or rs757228 polymorphisms. We also found that POAG patients had statistically significant (after correction for multiple testing) lower plasma vitamin E (p<0.001) and vitamin C (p<0.001) concentrations than control subjects. However, we detected a higher plasma GPx activity in POAG cases than in controls (p<0.001). The rs6994076 and rs1279683 polymorphisms were significantly (p<0.001) associated with plasma vitamin E and vitamin C, respectively. However, the rs757228 polymorphism in the *GPX4* gene was not associated with plasma GPx activity.

**Conclusions:**

We have described a novel association between the rs737723 polymorphism (*SEC14L2/TAP)* and higher POAG risk and confirmed the association between rs1279683 (*SLC23A2*) and POAG. Our results also suggested a gene–gene interaction between both polymorphisms that increases POAG risk.

## Introduction

Primary open-angle glaucoma (POAG) is a common optic neuropathy leading to progressive loss of vision and irreversible blindness if undetected and untreated [1]. Most of the molecular mechanisms leading to POAG development are still unknown, and although the risk factors of this pathology are still not fully identified, it is known that there is an important family aggregation that involves a strong genetic component [2,3]. Recent studies have described various genes related to greater POAG risk [4-9]; however, the associations vary depending on populations [8,10,11]. These differences could be related to other genetic characteristics of the different populations but also to environmental factors that differ between populations [12]. Recently some gene-environment interactions between the endothelial nitric oxide synthase gene variants and tobacco smoking [13], alcohol consumption [13], postmenopausal hormone use [14], or reproductive characteristics in women [15] have been reported in determining POAG risk. Among them, gene–diet interactions are of great interest given that diet can be considered as either a risk factor or a protective factor against POAG depending on the contribution of vitamins or other antioxidant compounds [16-18]. Although some epidemiological studies have shown that a higher fruit and vegetable intake is associated with lower POAG risk [19-21], there have been few studies addressing this, and the consistency level for specific nutrients is low [19-23]. This may be due to the fact that in these studies diet was measured by questionnaires but not by specific biomarkers. This is important as we know that nutrient status, i.e., vitamin concentrations, do not depend simply on their intake but on other genetic factors related to their absorption, transport, distribution, or metabolism [24].

Currently, studies on the nutrigenetics of POAG are scarce. Our group published one of the first works in this field [25] showing that a polymorphism (rs1279683) in the vitamin C co-transporter gene (a sodium L-ascorbic acid co-transporter [SVCT], isoform SVCT2, encoded by the solute carrier family 23 member 2 gene [*SLC23A2*]) was associated with lower plasma vitamin C concentrations and also with higher POAG risk. Continuing along this line, we are interested in other vitamin-related genes and oxidative stress parameters as oxidative stress plays a central role in glaucomatous pathogenesis [1,26,27]. α-Tocopherol, is the most active form of vitamin E and has high antioxidant capacity and other relevant functions [28-32]. There are several genes that participate in vitamin E metabolism. The tocopherol (α) transfer protein gene (*TTPA*) has been described as the major determinant of plasma α-tocopherol levels in humans [32,33]. The tocopherol associated protein (TAP), encoded by the SEC14-like 2 (*S.*

*cerevisiae*) gene (*SEC14L2*) gene may be specifically involved in the intracellular transport of tocopherol, e.g., between membrane compartments among other functions [34,35]. Despite the relevance of these genes, no study has evaluated whether genetic variation in these vitamin E-related genes is associated with glaucoma risk.

Another important antioxidant in the eye is glutathione peroxidase (GPx), a selenoprotein that protects cells from oxidative damage by reducing lipid peroxides. There are several isoenzymes encoded by different genes. GPx4 is one of these isoenzymes and is the only GPx isoenzyme that reduces phospholipid hydroperoxides [36]. Some studies have found that *GPX4* is needed for the maturation process of the retinal photoreceptors and protects the retina from oxidative damage [37,38]. However, the association between *GPX4* polymorphisms and POAG has not been investigated. Thus, our objectives were: 1) to confirm the association between the rs1279683 polymorphism in the *SLC23A*2 gene and POAG risk in a larger overlapping data set and to extend our findings by analyzing the differential expression of this gene in POAG cases and controls; 2) to investigate the effect of vitamin E-related polymorphisms (rs6994076 in the *TTPA* gene and rs737723 in the *SEC14L2/TAP* gene) on plasma vitamin E concentrations and POAG risk as well as the association between the rs757228 in the *GPX4* gene and GPx activity and POAG risk; 3) to study the joint contribution of these polymorphisms, including gene–gene interactions, on POAG risk in a Mediterranean population.

## Methods

### Subjects and study design

A matched case-control study was performed on both POAG and control subjects from a Mediterranean population. Subjects were paired by gender, age (±2 years), and body mass index (BMI; categorized into normal weight, overweight, and obese subjects). The BMI categories we used were: from 18.5 to less than 25 kg/m^2^ (normal weight, reference category), 25 kg/m^2^ to less than 30 kg/m^2^ (overweight), and 30 kg/m^2^ or greater (obesity). We recruited 500 Caucasian participants (250 patients with POAG, 106 men and 144 women, mean age 70±9 years; and 250 controls) from two health centers in the city of Valencia, Spain—the University Hospital Dr. Peset, Valencia (Department of Ophthalmology and Department of Preventive Medicine & Public Health) and the School of Medicine of the University of Valencia (Department of Preventive Medicine & Public Health) —so increasing the sample size of our first study in which we focused on the first 150 cases and 150 controls [25]. The Ethical Committees of the University of Valencia approved the protocols for this study, which complied with the Helsinki guidelines on human research. Informed consent forms were signed by all study participants.

Cases were subjects with POAG diagnosed by an ophthalmologist and were aged from 40 to 90 years. Diagnoses of glaucoma were determined when patients had at least two of the three following characteristics: 1) the appearance of the papilla: relationship cup–disc (CDR)>0.3 on the vertical axis or asymmetry of 0.2 in the eye engaged, thinning at the edge, or papillar bleeding (disc with suspected glaucoma); 2) intraocular pressure greater or equal to 22 mmHg (ocular hypertension); 3) visual field defect compatible with glaucoma: arcuate scotoma (or Bjerrum scotoma), nasal step, paracentral scotoma, general depression in the absence of other causes of these abnormalities (abnormal visual field). The optic nerve assessments were performed by means of ophthalmoscopy and optical coherence tomography (OCT) and were performed by ophthalmologists of our research group. The CDR was assessed using the data from cirrus OCT. To assess the optic nerve, we evaluated the width of the neuroretinal rim, the presence of papillary bleeding, the size of the excavation, and symmetry with the other eye. For the evaluation of the neuroretinal rim, we observed the inferior, superior, nasal, or temporal “(ISNT) rule” (I>S>N>T).

Controls were subjects living in the same geographical area without glaucoma, cataracts, age-related macular degeneration, or severe myopia (six or more diopters) and were matched with POAG cases by sex, age, and BMI. The controls were also examined by an ophthalmologist. Controls, as cases, did not have severe myopia. Subjects with severe myopia have an enlarged eyeball, posterior staphyloma, changes in the fibrillary network of the vitreous, chorioretinitis with choroidal atrophy plates, and degenerative lesions in the retinal periphery. However, to simplify matters we defined “severe myopia” as one in which subjects have six or more diopters.

Cases with ocular diseases other than POAG and/or rickets, osteoporosis and osteomalacia, and/or aged outside the range of inclusion were excluded. Controls with rickets, osteoporosis or osteomalacia, aged outside the range of inclusion, and/or with a family history of glaucoma were excluded. POAG patients underwent a systematized ophthalmic examination, including slit-lamp biomicroscopy, interocular pressure (IOP) measurement (with Goldmann Applanation Tonometry AT900, Haag-Streit, Bern, Switzerland), best corrected visual acuity, visual field performance (using the 24–2 Swedish Interactive Threshold Algorithm [SITA; Humphrey Field Analyzer II; Carl Zeiss Meditec, Inc., Dublin, CA]), ocular imaging analysis (using OCT [Stratus OCT; Carl Zeiss Meditec, Inc.]), and dilated stereoscopic fundus examination under a 78-D lens with simultaneous optic disc photographs. Data on duration of the disease, family background, therapy, and various aspects of the ophthalmological examination were registered using a self-designed database. IOP was also measured in healthy controls. POAG cases and controls completed a questionnaire regarding sociodemographic, clinical, and lifestyle variables.

### Biochemical and genetic analysis

Whole blood samples of both POAG and control groups were obtained from the antecubital veins of the subjects under fasting conditions (10–11 h). Blood was collected into EDTA tubes (three 5-ml tubes per subject, one for DNA extraction, another for RNA extraction, and the last for obtaining plasma).

Genomic DNA was extracted from blood with the MagNA Pure LC DNA Isolation kit (Roche Diagnostics, Mannheim, Germany). Genotyping of the following single nucleotide polymorphisms (SNPs) was performed on a 7900HT Sequence Detection System (Applied Biosystems, Foster City, CA) with standard TaqMan fluorescent allelic discrimination assays: the rs1279683 in the *SLC23A2* gene, rs6994076 in the *TTPA* gene, rs737723 in the *SEC14L2 (TAP)* gene, and rs757228 in the *GPX4* gene. We selected the rs1279683 (*SLC23A2*) polymorphism for its association with POAG risk in our previous study [25]. The rs6994076 polymorphism, located in the *TTPA* gene promoter (−980T>A) was chosen for its functional relevance, given that two previous studies had found significant associations between this polymorphism and plasma vitamin E concentrations [39,40]. For the *SEC14L2 (TAP)* and *GPX4* genes, no polymorphism has consistently been associated with vitamin E concentrations or GPx activity, respectively, in previous studies. For this reason we selected the rs737723 (intron 2 of the *SEC14L2* gene) and the rs757228 (−2050 G>A) in the 5′-untranslated region (5′-UTR) of the *GPX4* gene for their high minor allele frequency and as tag SNPs of the haplotype block of which they form part. In addition, we hypothesized that these polymorphisms may be candidates for a possible epigenetic regulation (by microRNAs, other noncoding RNAs, methylation) [41,42] for testing in later studies. Call rates for the genotyped SNPs were: 99.2% for rs1279683 in the *SLC23A2* gene, 96.8% for rs6994076 in the *TTPA* gene, 96.2% for rs737723 in the *SEC14L2/TAP* gene, and 97.2% for rs757228 in the *GPX4* gene.

RNA was obtained from the blood of 40 POAG patients selected at random and 40 controls using the extraction method with Trizol. Once the RNA samples were obtained, we proceeded to obtain cDNA from 300 ng of each sample by reverse transcription PCR, using the High Capacity RNA to cDNA Master Mix (Applied Biosystems). The expression of *SLC23A2* gene was analyzed by real-time PCR, using a 7900HT Sequence Detection System and TaqMan gene expression assays (Applied Biosystems). We assayed the samples in duplicate and used the *18S rRNA* gene as internal control. Results were expressed as fold changes in gene expression.

Plasma vitamin C concentrations were measured using the Li et al. method [43]. Analyses were performed using Shimadzu Scientific Instruments (SSI, Columbia, MD) equipment with an LC-20AB delivery pump and an electrochemical detector under reversed-phase conditions with a 4.6×250 mm, 5 µM YMC-Pack ODS-AQ column (Waters Corp., Milford, MA). The software used was LabSolutions 1.2 (SSI, Columbia, MD). Compounds were eluted over an 18-min runtime at a flow rate of 0.6 ml/min. The mobile phase consisted of methanol–150 mM chloroacetate (3:97, volume [v]/v) and 2 mM disodium EDTA (pH adjusted to 3.0 with 10 N NaOH). Sample injection was 5 µl.

Plasma vitamin E concentrations were determined by the Arnaud et al. method [44] using Shimadzu Scientific Instruments equipment with an LC-20AB delivery pump and a UV-Vis detector (290 nm) with a 4.6×250 mm, 5 µM YMC-Pack ODS-AQ column (Waters Corp.). The software used was LabSolutions 1.2 (SSI). Compounds were eluted over a 20-min runtime at a flow rate of 1.2 ml/min. The mobile phase consisted of acetonitrile–dichloromethane–methanol (72.5/22.5/5). Sample injection was 50 µl.

The plasma activity of glutathione peroxidase was determined using a commercial kit (Cayman Chemical Company, Ann Arbor, MI), measuring the decrease in absorbance at 340 nm produced by the oxidation of the reduced form of nicotinamide adenine dinucleotide phosphate (NADPH) to NADP+.

Vitamin C and vitamin E determinations were undertaken in duplicate for each individual, and a mean value of these measurements was used for each individual in the statistical analysis. The determination of the activity of GPx was performed in triplicate. Samples were not all run in one batch. We included POAG case and control samples in each batch and used random sampling of different batches to determine if there were any batch effects. We did not observe any differences in the results and so were able to discard batch effects. The mean values of intra-assay and inter-assay coefficients of variation were found to be 1.14% and 1.45%, respectively, for vitamin C; 1.79% and 1.94%, respectively, for vitamin E; and 2.09% and 2.21%, respectively, for GPx.

### Statistical analysis

Pearson’s chi-square test was used to compare categorical variables and to check Hardy–Weinberg equilibrium for the polymorphisms analyzed. The normal distribution of continuous variables was checked first. These variables were analyzed by the unpaired Student *t* test (two groups) or the analysis of variance test (for more than two groups). Given that the frequency of the polymorphisms analyzed may be different in different geographical areas and to avoid confusion in the so-called “minor allele”, we used the criterion of The National Human Genome Research Institute circulated in the Single Nucleotide Polymorphism Database (dbSNP), which determines the ancestral alleles for human SNPs by the comparison of human DNA to chimpanzee DNA [45]. For the four polymorphisms analyzed, we coded the ancestral allele as 1 and the nonancestral allele as 2. Having not observed any clear additive effects of the alleles in their association with POAG risk, we performed the genetic analyses by grouping homozygous and heterozygous subjects for the ancestral allele together and comparing them with homozygous subjects for the nonancestral (variant) allele (dominant or recessive effects depending on the frequency of the minor allele). In addition, in the comparison of mean values of vitamins depending on the genotypes, we also considered an additive model (variant alleles coded as 0, 1, and 2) for the rs6994076 polymorphism and the linear regression coefficient (B) was estimated.

To estimate the risk of glaucoma associated with the genotypes, logistic regression models were fitted, and the odds ratios (OR) and their 95% confidence intervals (CI) were estimated. Multivariate regression models were also fitted, including control for potential confounders: sex, age (as continuous), BMI (as continuous), tobacco smoking (coded as current smoker or not), and alcohol consumption (current drinker or not). Multivariate linear regression models (for continuous variables, including vitamin concentrations and GPx activity) or logistic regression models (for POAG risk) were used to adjust the associations for potential confounders. In addition, hierarchical regression models (linear and logistic) with main effects and interactions terms as well as with adjustment for potential confounders were used to study the gene–gene interactions in determining POAG risk or vitamin concentrations. All probability values were based on two-tailed tests. The original significance level was set at a p value of 0.05 (nominally significant) by a two-tailed test, with the Bonferroni correction applied to compensate for multiple comparisons. This approach consists of using an adjusted α level equal to the original α level (0.05), divided by the number of SNPs analyzed (four). In our case, the adjusted α is 0.05/4=0.0125. The significance of each uncorrected test was assessed at this level. PASW software (version 17.0, SPSS, Inc., Chicago, IL) and the program Haploview 3.32 (Broad Institute of MIT and Harvard, Boston, MA) were used for statistical analysis.

## Results

**Table 1** shows the sociodemographic characteristics of all 250 POAG patients and their 250 paired controls. In addition to sex and age, we matched cases and controls by BMI (categorized into normal weight, overweight, and obese subjects) because plasma vitamin C concentrations and other antioxidants are associated with BMI [46], thus minimizing the likelihood of confounding by BMI. Genotype distributions of all SNPs in the POAG cases and controls exhibited Hardy–Weinberg equilibrium.

**Table 1:** General characteristics of primary open glaucoma cases and controls

**Characteristics**		**Cases (n=250)**	**Controls (n=250)**	**p**
Females (%)		57.6	57.6	1.000
Age (years)		70.1±8.7	70.0±8.6	0.987
BMI (kg/m^2^)		27.3±4.1	27.2±4.1	0.823
Cup disk ratio		0.7	0.3	<0.001
Smokers* (%)		30	24	0.159
Alcohol consumers** (%)		66	58	0.065

BMI: Body mass index *Current smokers **Current drinkers

### Association between the selected polymorphisms and primary open-angle glaucoma risk

The genotype frequencies of the four SNPs in POAG cases and controls are shown in **Table 2**. For each SNP we have first indicated the genotypes for the ancestral allele [45], which was the major allele for the rs757228 polymorphism in the *GPX4* gene and the rs737723 polymorphism in the *SEC14L2/TAP* gene (controls). In this Mediterranean population, the ancestral allele was the minor allele for the other two SNPs. We grouped homozygous and heterozygous subjects for the ancestral allele and compared this group (reference category) with homozygous subjects for the nonancestral (variant) allele. Crude and multivariate adjusted p values were estimated (Table 2). We observed a statistically significant (p<0.0125, after correction for multiple testing) association between the rs1279683 polymorphism in the vitamin C co-transporter (*SLC23A*2) and POAG risk (unadjusted OR 1.70, 95% CI 1.17–2.47, p=0.005) for GG homozygotes in comparison with carriers of the A allele after correction for multiple comparisons. This association remained statistically significant after correction for multiple testing even after adjustment for sex, age, BMI, alcohol, and tobacco smoking. This result confirmed our previous findings in a subsample of 150 POAG cases and 150 controls [25], so adding consistency to the association. We also obtained a new association between the rs737723 polymorphism in the *SEC14L2/TAP* gene and POAG risk (unadjusted OR 1.78, 95% CI 1.18–2.69, p=0.006) for CC homozygotes in comparison with G allele carriers. This associations remained statistically significant (p<0.0125) following multivariate adjustment for sex, age, BMI, alcohol, and tobacco smoking (adjusted OR 1.73, 95% CI 1.13–2.65, p=0.011) and correction for multiple comparisons. The polymorphisms in the *TTPA* and *GPX4* genes were not significantly associated with POAG.

**Table 2:** Genotypic frequencies of the *SLC23A2, TTPA, TAP* and *GPX4* SNPs in primary open glaucoma (POAG) cases and controls and association with POAG risk. Crude and multivariate estimations

**Genes**	**SNP**		**Genotype frequencies (%)**						Independent analysis by SNP					Multivariate model with all SNPs	
		Alleles*	**Cases**			**Controls**									
		**(1/2)**	**1/1**	**1/2**	**2/2**	**1/1**	**1/2**	**2/2**	**p1**	OR** (95%CI)	**p2**	OR *** **(95% CI)**	**p3**	**OR^ (95%CI)**	**p4**
*SLC23A2*	rs1279683	A/G	11.7	47.4	40.9	16.9	54.2	28.9	0.014	1.70 (1.17–2.47)	0.005	2.11 (1.34–3.32)	0.001	2.47 (1.62–3.77)	<0.001
*TTPA*	rs6994076	T/A	22.0	46.5	31.5	22.2	49.8	28	0.673	1.19 (0.80–1.75)	0.393	1.17 (0.79–1.75)	0.429	1.38 (0.92–2.10)	0.122
*TAP/SEC14L2*	rs737723	G/C	19.9	47.6	32.5	28.5	50.2	21.3	0.009	1.78 (1.18–2.69)	0.006	1.73 (1.13–2.65)	0.011	2.24 (1.44–3.48)	<0.001
*GPX4*	rs757228	G/A	29.9	50.0	20.1	24	53.3	22.7	0.325	0.85 (0.55–1.32)	0.477	0.82 (0.52–1.28)	0.371	0.80 (0.50–1.27)	0.337

SNP: Single nucleotide polymorphism *Allele 1 is the ancestral allele according to the National Center for Biotechnology Information Database (dbSNP). Allele 2 is the variant allele **: Unadjusted odds ratio (OR) and 95% confidence interval (CI) for homozygous subject for the variant allele [2] in comparison with the reference category (homozygotes and heterozygotes for the ancestral allele) for each SNP in the separate genetic analyses. Separate model for each SNP; ***: OR adjusted for sex, age, BMI, smoking and drinking did not change the statistical significance. ^: OR for each SNP obtained in the multivariate model including at the same time the four SNPs analyzed. Further adjustment for sex, age, BMI, smoking and drinking did not change the statistical significance. p1: p value obtained in the corresponding Chi-square test for the comparison of the genotype distribution between cases and controls (three groups) p2: p value obtained in the crude logistic regression analysis for the OR for each SNP in the individual genetic analyses p3: p value in obtained in the multivariate logistic regression after adjustment for sex, age, BMI, tobacco smoking and drinking for each SNP in the individual genetic analysis. p4: p value obtained in the crude logistic regression analysis for the OR for each SNP in the multivariate genetic analysis including all SNPs in the multivariate model. Presented p values are uncorrected for multiple comparisons. Considering the correction for multiple comparisons, an uncorrected p value <0.0125 was considered to be statistically significant

We also examined the joint contribution of the polymorphisms studied in a multivariate regression model, including all of them together (Table 2), and found that both the rs1279683 (*SLC23A*2) and the rs737723 (*SEC14L2/TAP*) polymorphisms maintained the statistical significance after correction for multiple comparisons and even increased the magnitude of their association with POAG risk in the multivariate model in comparison with their association when using separate models. The rs6994076 polymorphism in the *TTPA* gene also increased the magnitude of the association but did not the reach statistical significance. After adjustment of this model for sex, age, BMI, smoking, and drinking, the statistical significance of the polymorphisms did not vary, and both the rs1279683 (*SLC23A*2) and the rs737723 (*SEC14L2/TAP*) polymorphisms remained significantly associated (p<0.0125, after correction for multiple testing) with POAG risk (adjusted OR 3.20, 95% CI 1.94–5.29, p<0.001 for the rs1279683 polymorphism and adjusted OR 2.22, 95% CI 1.41–3.51, p=0.001 for the rs737723 polymorphism).

Moreover, we found a nominally significant (p=0.047) gene–gene interaction between the rs1279683 polymorphism in the *SLC23A2* gene and the rs737723 polymorphism in the *SEC14L2/TAP* in determining POAG risk. When subjects were homozygotes for the GG genotype of the *SLC23A2* and for the CC genotype of the *SEC14L2/TAP* simultaneously, the interactive effect of the joint presence of these polymorphisms multiplied POAG risk. Thus, in the stratified analysis by genotypes, the risk of POAG for subjects with the GG genotype of the rs1279683 (*SLC23A2*) polymorphism in subjects having at the same time the CC genotype for the rs737723 polymorphism in the *SEC14L2/TAP* gene (4.2% of the whole sample) increased several times (unadjusted OR 14.8, 95% CI 1.93–113.7, p=0.009) and remained statistically significant after correction for multiple testing (p=0.006) following adjustment for age, sex, BMI, smoking, and drinking. However, in subjects carrying the G allele (CG or GG) for the *SEC14L2/TAP* gene, the association between the rs1279683 (*SLC23A2*) polymorphism and POAG risk was lower (unadjusted OR 1.77, 95% CI 1.11–2.84, p=0.016). This association was statistically significant after correction for multiple testing (p=0.001) and adjustment for age, sex, BMI, smoking, and drinking.

### Plasma concentrations of vitamin C, vitamin E, and glutathione peroxidase activity in both primary open-angle glaucoma cases and controls. Influence of the selected polymorphisms

We also analyzed the plasma concentrations of vitamin C, E, and GPx in POAG cases and controls and found lower statistically significant (after correction for multiple testing) plasma concentrations of both vitamins (vitamin C, 10.0±1.6 µg/ml versus 12.0±1.7 µg/ml, p<0.001; vitamin E, 10.7±1.7 µg/ml versus 11.4±1.8 µg/ml, p<0.001) in POAG cases than in controls. However, we observed a higher GPx activity in POAG patients than in control subjects (26.3±8.5 U/ml versus 17.7±6.2 U/ml, p<0.001, respectively).

On analyzing the associations of the polymorphisms with these biomarkers (**Table 3**), we also observed that the GG genotype in the *SLC23A2* gene was associated with lower plasma concentrations of vitamin C (after correction for multiple testing), so replicating the association previously described in our previous study [25]. Furthermore, the AA genotype for the rs6994076 polymorphism in the *TTPA* gene was significantly (after correction for multiple testing) associated with lower plasma levels of vitamin E. However, the rs737723 polymorphism in the *SEC14L2/TAP*, which presented a significant association with POAG risk (after correction for multiple testing), was not significantly associated with lower plasma vitamin E concentrations in the whole population. These results did not change in statistical significance after adjustment for sex, age, BMI, smoking, and drinking. Given that this polymorphism presented a gene–gene interaction with the rs1279683 polymorphism in the *SLC23A2* gene in determining POAG risk, we also studied this gene–gene interaction in determining vitamin E concentrations. We observed that in POAG cases (not in controls), the rs737723 polymorphism in *SEC14L2/TAP* was associated at the nominal level (p<0.05) with lower plasma vitamin E concentrations (10.0±0.35 µg/mL in CC versus 10.9±0.17 µg/ml in CG or GG subjects for the rs737723; p=0.045) only in the subgroup of POAG cases with the GG genotype for the rs1279683 polymorphism in the *SLC23A2* gene. This GG genotype, in turn, was associated with lower vitamin C concentrations. Differences in plasma vitamin C and E levels between POAG subjects and controls were within the normal range for mean levels. We did not observe any significant interaction between the rs1279683 polymorphism in the *SLC23A2* gene and the rs6994076 polymorphism in the *TTPA* gene. The SNP in the rs757228 in the *GPX4* gene was not associated with GPx activity (Table 3).

**Table 3:** Plasma vitamin C, vitamin E and glutathione peroxidase concentrations (mean ± SD) depending on the corresponding *SLC23A2, TTPA, TAP or GPX4* genotypes in primary open glaucoma cases and controls

**Vitamin**	**Gene**	**SNP**	Alleles*	**Plasma concentration of antioxidants**									
				**Cases (n=250)**					**Controls (n=250)**				
			**(1/2)**	**1/1**	**1/2**	**2/2**	**p1**	**p2**	**1/1**	**1/2**	**2/2**	**p1**	**p2**
**Vitamin C** (µg/ml)	*SLC23A2*	rs1279683	A/G	10.5±1.7	10.6±1.4	9.1±1.5^a,b^	<0.001	<0.001	12.5±1.7	12.5±1.4	10.6±1.4^a,b^	<0.001	<0.001
**Vitamin E** (µg/ml)	*TTPA*	rs6994076	T/A	11.1±1.5	11.0±1.6	10.1±1.8^c^	<0.001	<0.001	12.0±1.5	11.5±1.7	10.6±2.0^d^	<0.001	<0.001
**Vitamin E** (µg/ml)	*TAP*	rs737723	G/C	10.6±1.6	11.0±1.7	10.5±1.7	0.103	0.095	11.3±1.7	11.4±1.9	11.4±1.7	0.984	0.997
**GPx** (U/ml)	*GPX4*	rs757228	G/A	25.9±8.7	26.8±8.0	26.0±9.5	0.727	0.752	18.8±7.0	17.8±6.1	16.5±5.2	0.146	0.094

GPx: glutathione peroxidase; SD: Standard deviation; SE: Standard error. *Allele 1 is the ancestral allele. Allele 2 is the variant allele. p1: p value obtained in the ANOVA test including the three genotypes. p2: p value obtained in the Student t test comparing the homozygous subjects for the variant allele [2] with carriers of the ancestral allele (1/1+1/2) for each polymorphism. Post-hoc comparisons: a: p values for comparison of means of vitamin C concentrations in GG subjects versus AA (p<0.001 in POAG cases and p=0.001 in controls); b: p values for comparison of means between vitamin C concentrations in GG subjects versus AG (p<0.001 in POAG cases and p=0.001 in controls). c: Regression coefficient (B) ±SEM and p value for additive effects (coded a 0, 1 or 2 variant alleles, A) in POAG cases (B=-0.54±0.14 µg/ml; p<0.001). d: Regression coefficient (B) ±SEM and p value for additive effects (coded a 0, 1 or 2 variant alleles, A) in controls (B=-0.70±0.16 µg/ml; p<0.001). A p value <0.0125 indicate that the results is statistically significant after correction for multiple testing.

### Gene expression analysis

Finally, we studied the expression of the *SLC23A2* gene in 40 POAG patients and 40 control subjects for whom RNA was available in order to better characterize the results of our previous study. We observed a significantly (after correction for multiple testing) lower expression (fold change) of the *SLC23A2* gene in POAG cases in comparison with the control group (0.97±0.04 versus 1.00±0.03, p<0.001, respectively). These results support the implication of lower *SLC23A2* activity in determining lower vitamin C concentration in POAG cases than in controls.

## Discussion

This is the first study to examine whether common variants in two key genes involved in vitamin E transport (*TTPA* and *SEC14L2/TAP*) are associated with POAG risk. We found for the first time a statistically significant association (after correction for multiple testing) between the rs737723 polymorphism in the *SEC14L2/TAP* gene and higher POAG risk in CC homozygotes in comparison with carriers of the G allele. In addition, we confirmed the association between the rs1279683 polymorphism in the *SLC23A*2 gene and higher POAG risk, which we reported in a subsample of the present study [25]. Why no previous association has been detected in these loci in genome-wide association studies (GWAs) with greater sample sizes may be due to various reasons, among them being that these loci were not well represented in the GWAs and the presence of interaction with environmental factors. Moreover, it is well known that GWAs are not totally efficient because of type-2 errors that are made when requiring a very low p value to consider an association as statistically significant at the GWA level.

In our study, we also confirmed the association of the rs1279683 polymorphism with plasma vitamin C concentrations [28]. These associations occur consistently, given that the genotype that is associated with greater POAG risk (homozygous subjects for the G allele) is also the genotype that is associated with lower plasma vitamin C concentrations even after correction for multiple testing. Although the importance of the *SLC23A2* gene in the transport and metabolism of vitamin C has been confirmed by numerous studies [47,48], none of them focused on POAG risk. Moreover, unlike the SVCT1 transporter, the SVCT2 (encoded by the *SLC23A2* gene) is expressed in a greater number of tissues, including the eye [48,49]. In the present study, we have also reported for the first time that *SLC23A2* gene expression in peripheral blood cells is significantly lower in POAG cases than in controls, thereby suggesting a direct relationship between gene expression and plasma vitamin C concentrations. These results and their effects on POAG risk are also consistent with other studies performed on mice demonstrating that low vitamin C and increased oxidative stress and cell death in mice that lack the sodium-dependent vitamin C transporter SVCT2 [50] (or also that an increase in the expression of the *SVCT2* gene in the mouse model) raises ascorbic acid in tissues and protects against paraquat-induced oxidative damage [51].

Our results also suggested a gene–gene interaction between the rs1279683 polymorphism in the *SLC23A2* gene and the rs737723 in a vitamin E transporter gene (*SEC14L2/TAP*), so increasing POAG risk. Although the interaction term was only nominally significant, in the stratified analysis by polymorphism we observed statistically significant results even after correction for multiple comparisons. Thus, in subjects who were simultaneously homozygous for the risk genotype of both polymorphisms (GG for the rs1279683 and CC for the rs737723 polymorphisms), POAG risk increased more than sevenfold. This is an interesting interaction as it may help us to better understand the boosting mechanisms of the effects of low concentrations of vitamin C and vitamin E on oxidative stress or on other effects of these genes, taking into account that the TAP protein seems to have multiple biologic functions, including that of mediator of vitamin E-dependent gene expression [28,29,52,53]. Likewise, it helps to explain the influence of the rs737723 (*SEC14L2/TAP*) polymorphism on plasma vitamin E concentrations as, despite obtaining a strong association of this polymorphism with greater POAG risk, we did not find a statistically significant association of this polymorphism with vitamin E concentrations in the overall analysis. Thus, despite not having found such association in the whole population, in the stratified analysis we did find that in patients with POAG, the effect of the rs737723 polymorphism on vitamin E concentrations was modulated by the rs1279683 polymorphism in the *SLC23A*2 gene. Hence, in homozygous subjects for the genotype associated with lower vitamin C concentrations (GG), rs737723 (*SEC14L2/TAP)* was nominally associated with lower vitamin E concentrations. Although this association was not statistically significant after correction for multiple comparisons, it is highly consistent with the interaction effect that we observed between both polymorphisms in determining POAG risk. Possibly the influence of the rs737723 polymorphism (*SEC14L2/TAP)* cannot be explained just by its regulation of vitamin E concentrations, and there may be other mechanisms involved, such as the modulation of the expression of other genes [29,35]. Currently, the function of the *SEC14L2/TAP* gene is not well known [35], and further research is required.

Previous studies showed that the main determinant of vitamin E concentrations was the *TTPA* gene and not the *TAP* gene [28,29,32,33]. With regard to this, rare mutations in the *TTAP* gene are known to be the cause of a progressive neurodegenerative disorder known as ataxia with vitamin E deficiency. Despite normal dietary intake of vitamin E, affected individuals suffer from deficiency of this essential vitamin [33]. Our results are consistent with this previous knowledge as we have found a strong association between the rs6994076 polymorphism in the *TTPA* genes and vitamin E concentrations, thus replicating the previous findings of another group analyzing the same polymorphism and plasma vitamin E concentrations [39]. Paradoxically, we did not find any association between the rs6994076 polymorphism with POAG risk. Perhaps one possible explanation is that *TTPA* is mainly expressed in the liver, whereas *SEC14L2/TAP* is expressed in a greater number of tissues [28,29], thus suggesting a greater repercussion on intracellular vitamin E transport and effects at the ocular level. Finally, as regards the *GPX4* polymorphism, we did not find any association either with POAG risk or with GPx activity. However, we found statistically significant differences between GPx activity in cases and controls. Considering that we have only studied one polymorphism related to GPx activity, future efforts should concentrate on other relevant genetic variants. Although there are various studies supporting the hypothesis that antioxidant enzymes, such as GPx, have an influence on glaucoma risk, there is a discrepancy over whether these enzymes are increased or diminished in POAG cases [54,55]. In our study we found an increase (statistically significant after correction for multiple testing) in GPx in POAG cases supporting the hypothesis that, due to the fact that antioxidant defenses are decreased in patients with glaucoma, the activity of antioxidant enzymes is increased in an attempt to counteract the damage caused by reactive oxygen species.

In conclusion, we report for the first time a significant association between the rs737723 polymorphism in the *SEC14L2/TAP* gene and POAG risk and confirmed the previously reported association between the rs1279683 polymorphism in the *SLC23A2* gene with POAG risk. Moreover, our results suggested a gene–gene interaction between both polymorphisms, so increasing the POAG risk in subjects who simultaneously have both risk genotypes. This effect may also be mediated with the joint contribution of both polymorphisms in determining vitamin C and vitamin E concentrations in a subgroup of individuals. However, other additional mechanisms may be involved, and this needs to be investigated in new nutrigenetic studies. Despite the polymorphism in *TTPA* being associated with lower vitamin E concentrations, we did not observe its association with greater POAG risk.
